# Nitrogenase diversity and activity in the gastrointestinal tract of the wood-eating catfish *Panaque nigrolineatus*

**DOI:** 10.1038/ismej.2015.65

**Published:** 2015-04-24

**Authors:** Ryan McDonald, Fan Zhang, Joy E M Watts, Harold J Schreier

**Affiliations:** 1 Department of Biological Sciences, University of Maryland Baltimore County, Baltimore, MD, USA; 2 Institute of Marine and Environmental Technology, University of Maryland Center for Environmental Science, Baltimore, MD, USA; 3 School of Biology, University of Portsmouth, Portsmouth, UK; 4 Department of Marine Biotechnology, Institute of Marine and Environmental Technology, University of Maryland Baltimore County, Baltimore, MD, USA

## Abstract

The Amazonian catfish, *Panaque nigrolineatus*, consume large amounts of wood in their diets. The nitrogen-fixing community within the gastrointestinal (GI) tract of these catfish was found to include *nifH* phylotypes that are closely related to *Clostridium* sp., Alpha and Gammaproteobacteria, and sequences associated with GI tracts of lower termites. Fish fed a diet of sterilized palm wood were found to contain *nifH* messenger RNA within their GI tracts, displaying high sequence similarity to the nitrogen-fixing *Bradyrhizobium* group. Nitrogenase activity, measured by acetylene reduction assays, could be detected in freshly dissected GI tract material and also from anaerobic enrichment cultures propagated in nitrogen-free enrichment media; *nifH* sequences retrieved from these cultures were dominated by *Klebsiella-* and *Clostridium-*like sequences. Microscopic examination using catalyzed reporter deposition-enhanced immunofluorescence revealed high densities of nitrogenase-containing cells colonizing the woody digesta within the GI tract, as well as cells residing within the intestinal mucous layer. Our findings suggest that the *P. nigrolineatus* GI tract provides a suitable environment for nitrogen fixation that may facilitate production of reduced nitrogen by the resident microbial population under nitrogen limiting conditions. Whether this community is providing reduced nitrogen to the host in an active or passive manner and whether it is present in a permanent or transient relationship remains to be determined. The intake of a cellulose rich diet and the presence of a suitable environment for nitrogen fixation suggest that the GI tract microbial community may allow a unique trophic niche for *P. nigrolineatus* among fish.

## Introduction

The ability to consume and subsist on mature structural wood (‘xylophagy') is a rare dietary strategy among vertebrates. While it has been described in several taxonomically diverse groups of invertebrates, xylophagy has not yet been observed in any vertebrate system (Bridges, 1981; Gallager *et al.*, 1981; Scrivener *et al.,* 1989). Few terrestrial (for example, beavers and porcupines) and aquatic vertebrates have been shown to consume wood as part of their normal diet (Vispo and Hume, 1995; Armbruster, 2003), but, it is unclear whether it serves as an essential source of dietary carbon. The relative dearth of vertebrate xylophages is due in part to the limited nutritive value of wood. Although rich in carbohydrates, the predominant polysaccharide, cellulose, is highly recalcitrant to enzymatic hydrolysis and often requires the concerted efforts of mixed microbial communities for efficient saccharification.

In addition being difficult to digest, wood is nitrogen deficient, with carbon to nitrogen ratios well above those found for the tissues of primary consumers (Mattson, 1980). All xylophagic systems characterized so far, possess an obligate enteric diazotrophic microbial community (Eutick *et al.,* 1978; Lechene *et al.,* 2007). However, the nature of these symbioses vary greatly, ranging from single species housed in specialized tissue that is characteristic of wood-boring mollusks (*Teredinidae*) (Waterbury *et al.,* 1983; O'Connor *et al.,* 2014) to complex mixed microbial communities colonizing the anaerobic regions of the hindgut, which occurs in termite systems (*Isoptera*) (Ohkuma *et al.,* 1996; Ohkuma *et al.,* 1999). Historically, these communities were not well-characterized due to the inability to culture environmental isolates. However, with the advent of molecular techniques and next generation sequencing it has become clear that diazotrophic microorganisms have a critical role in the nutrition of many symbiotic organisms (Sabree and Moran, 2014). Given that nitrogen fixation has been observed in taxonomically disparate microbial species (Zehr *et al.,* 2003), it is not surprising that diazotrophic community composition varies between hosts. The most extensively characterized diazo-trophic communities are found in termites, which are traditionally thought to be dominated by *nifH*-expressing *Clostridia* spp., spirochetes and methanogens (Ohkuma *et al.,* 1999; Lilburn *et al.,* 2001; He *et al.,* 2013). However, recent analysis has revealed that the alternative nitrogenase (*anf*)-expressing *Bacteroidales* ectosymbionts of gut flagellates are primarily responsible for nitrogen fixation in the hindgut of dry wood-feeding termites (Noda *et al.,* 1999; Desai and Brune, 2012).

The Amazonian catfish, *Panaque nigrolineatus*, is one of two fish species known to consume large amounts of wood in their diets (Schaefer and Stewart, 1993). A member of the family *Loricariidae*, *P. nigrolineatus* appears uniquely adapted to utilize allochthonous carbon sources. It lacks the slender, flexible, hypomineralized teeth characteristic of *Loricariidae*, and instead possesses rigid, unicuspid teeth that have increased capacity for wood gouging (Geerinckx *et al.,* 2007; Geerinckx *et al.,* 2012). In addition, wood-eating *Loricariidae* have unique jaw structure and increased musculature, which includes increased adductor mandibulae muscle force (Lujan and Armbruster, 2012). Coupled with the reduced tooth count and increased tooth–jaw attachment strength, this allows wood-eating species to impart increased lateral shearing force on woody substrates, and increases the rate of wood consumption.

In contrast to their specialized teeth and jaw, *Panaque* do not appear to have a GI tract well adapted for wood digestion. The gastrointestinal (GI) tract is extremely long (~11 times the standard length of the fish) and thin-walled (German, 2009); an extended gut region characteristic of xylophagic and herbivorous animals that serves as the primary site for saccharification and fermentation of plant structural polysaccharides is lacking. Furthermore, the fish possess a relatively rapid gut transit time (~4 h) with minimal axial mixing of digesta (German, 2009). Although these characteristics are more akin to detritivorous organisms, rapid gut transit times have been observed in at least one other well-characterized herbivorous animal (Dierenfeld *et al.,* 1982) and may indicate a recent evolutionary shift in diet.

In addition to its unique morphology, *P. nigrolineatus* possesses a novel enteric microbial community that appears adapted for efficient cellulose degradation (McDonald *et al.,* 2012; Watts *et al.,* 2013). A combination of 16S ribosomal RNA (rRNA) gene sequencing and cellulose enrichment culturing identified several cellulolytic species belonging to the *Cytophaga*, *Clostridium*, *Bacteroides* and *Cellulomonas* genera. The microbial diversity detected was distinct from those observed on ingested wood and rearing water, suggesting that celluloytic microbial populations are selectively retained within different regions of the GI tract (McDonald *et al.,* 2012; Watts *et al.,* 2013). These microbial species are not unique to *P. nigrolineatus* and have been shown to play roles in cellulose degradation in xylophagic and herbivorous systems including wood-feeding beetles, silk worms and humans (Hemmat and Emtiazi, 2000; Robert and Bernalier-Donadille, 2003). Furthermore, a microbial community enriched for cellulose degraders was also found in feces from *P. nigrolineatus* (Di Maiuta *et al.,* 2013).

In comparison to the cellulose degrading capabilities within the *P. nigrolineatus* GI tract, the enteric diazotrophic diversity remains largely uncharacterized. Previous community analysis revealed that several putative diazotrophic members of the Alphaproteobacteria were present in high abundance in the anaerobic mid and hindgut (McDonald *et al.,* 2012) GI tract regions. The Alphaproteobacteria class included prolific nitrogen fixers, particularly Rhizobiales species found in association with plants, but as yet, not identified as a significant component of any enteric microbial community observed in fish (Wu *et al.,* 2010). However, community analysis in *Tetraponera*, which also consume a nitrogen deficient diet of plant exudates, suggest they are capable of forming animal symbioses and contributing to host nutrition (Van Borm *et al.,* 2002; Stoll *et al.,* 2007).

The purpose of this study was to characterize the enteric diazotrophic community of *P. nigrolineatus* and assess its ability to fix nitrogen *in situ*. Our findings suggest that the *P. nigrolineatus* GI tract provides a suitable environment for nitrogen fixation and in combination with its xylivorous ability indicates that *P. nigrolineatus* occupies a unique trophic niche among fish.

## Materials and methods

### Fish rearing conditions

Wild-caught *P. nigrolineatus* (L-190) were imported from South America by the fish wholesaler Aquascapeonline (Belleville, NJ, USA). Fish were housed individually in aerated tanks kept at 29±1 °C; fish <40 mm (standard length) were excluded from the analysis. Three weeks prior to the introduction of experimental diets, fish were fed an acclimation diet composed of hearts of palm (*Euterpe precatoria*), algae pellets (Hikari Tropical Sinking Algae Wafers, Hayward, CA, USA), and date palm wood (*Phoenix dactylifera*) specified by IACUC 071509JW-01. After this acclimation period, fish were provided with either a mixed diet of palm hearts and algae (which were provided every second day) along with wood or a wood-only diet, where wood was constantly available. For both acclimation and feeding experiments, wood was thoroughly soaked in water and autoclaved three times prior to placement in the tank. In addition to sterilizing the wood, this procedure facilitated water saturation, enabling the wood to sink to the bottom of the tank making it accessible to the fish. Fish were reared under dark/low light conditions to inhibit growth of algae. This feeding treatment was maintained for 3 weeks, which was concluded by dissection after anesthetic overdose in 3-aminobenzoic acid ethyl ester (MS-222, 50 mg l^−1^), as described previously (McDonald *et al.,* 2012).

### Tissue preparation

For *nifH* cloning and reverse transcriptase–PCR (RT–PCR) analysis, intact GI tracts of wood-fed fish were dissected on the benchtop over ice. Tissue samples were flash frozen and stored at −80 °C prior to nucleic acid extraction as described below.

For acetylene reduction assays and nitrogen-free enrichment cultures, killed wood- and mixed diet-fed fish were immediately placed in an inflatable glove bag (Glass-Col, Terre Haute, IN, USA), which was sealed, evacuated of air and re-inflated with N_2_ gas (zero grade); residual oxygen was removed by the addition of a steel wool O_2_ sink saturated with an acidified copper sulfate solution (Rosenblatt and Stewart, 1975). The presence of a reduced atmosphere was verified using methylene blue anaerobic indicator strips (BBL Microbiology Systems, Cockeysville, MD, USA). The GI tract was divided into three equal lengths representing fore, mid and hindgut sections and immediately used to establish anaerobic enrichment cultures and for use in acetylene reduction assays as described below.

### *nifH* cloning and RT–PCR

Frozen tissue samples from wood-fed fish were processed using the Roche High Pure Template Preparation Kit (Indianapolis, IN, USA) as described by the manufacturer, except for a modification that integrated both animal and bacterial DNA extraction procedures, which included the incubation of tissue in 200 μl of the kit's tissue lysis buffer and 40 μl proteinase K solution for 1 h at 55 °C. Microbial *nifH* genes were amplified using a nested primer procedure as described previously (Zehr and Turner, 2001; Jenkins *et al.,* 2004). The first round of amplification was performed using primers nifH32f (5′-TGAGACAGATAGCTATYTAYGGHAA-3′) and nifH623r (5′-GATGTTCGCGCGGCACGAADTRNATSA-3′) with an initial denaturation step of 5 min at 94 °C followed by 30 cycles of denaturation for 1 min at 94 °C, annealing for 1 min at 50 °C, elongation for 1 min at 72 °C, followed by a final elongation for 10 min at 72 °C. The second amplification was performed using primers nifH1 (5′-TGYGAYCCNAARGCNGA-3′) and nifH2 (5′-ADNGCCATCATYTCNCC-3′) with an initial denaturation step of 3 min at 94 °C followed by 35 cycles of denaturation for 1 min at 94 °C, annealing for 1.5 min at 54 °C, elongation for 1.5 min at 72 °C, followed by a final elongation for 10 min at 72 °C. The second primer set amplifies the *Azotobacter vinelandii nifH* coding region (GenBank accession M20568) from positions 115–476. The size (~350 bp) and yield of PCR products were verified by gel electrophoresis. Amplified products from the second round of PCR were ligated into vector pCR2.1 (Invitrogen, Carlsbad, CA, USA) according to the manufacturer's instructions and ligation products were transformed into chemically competent One Shot Top10 *Escherichia coli* (Invitrogen) and transformants were selected on LB agar with ampicillin (50 μg ml^−1^) and X-gal (40 μg ml^−1^). Transformants harboring plasmids with inserts were collected and DNA sequence was determined by the Institute of Marine and Environmental Technology BioAnalytical Services Laboratory using an ABI 3130 XL Genetic Analyzer (Life Technologies, Grand Island, NY, USA). Sequences were deposited in GenBank with accession numbers KF025332-KF025377.

RNA for RT–PCR was extracted from GI tract sections of wood-only fed fish using the PureLink RNA kit from Invitrogen following the manufacturer's instructions. RNA (3 μg) was first treated with DNaseI (Fermentas, Hanover, MD, USA) in the presence of RNasin (Promega, Madison, WI, USA) and RT–PCR was carried out using the Superscript III One-Step Platinum Taq high fidelity kit (Invitrogen) with a 15 min complementary DNA synthesis step at 55 °C followed by amplification with nifH32 and nifH623 primers as described above. A second round amplification was done using nifH1 and nifH2 primers and *Taq* polymerase mix from Qiagen (Valencia, CA, USA) as described above. Reaction products were separated on a 1.5% agarose gel. Cloning and sequencing reactions were prepared from the RT–PCR product as described above.

### Enrichment cultures

Dissected tissue regions were prepared (as described above) and each was subsequently divided into four equal length sections, combining the first and third of each region and homogenizing in 5 ml of enrichment media using a glass tissue grinder (Pyrex, Corning, NY, USA). The remaining two sections of each region were used to inoculate 10 ml of nitrogen-free enrichment medium (Potrikus and Breznak, 1977), which was sealed and maintained anaerobically in 20 ml Hungate tubes (VWR, Radnor, PA, USA) with gas-tight septa (Miller and Wolin, 1974). Cultures were incubated at 25 °C at 190 r.p.m. for 4 days. Enrichment cultures were transferred to fresh media anaerobically in a glove bag by injecting 100 μl of inoculum through the septum and incubated for an addition 4 days followed by one additional passage. Total microbial DNA was extracted from enrichment cultures and community *nifH* genes were amplified according to the methods described above. Sequences were deposited in GenBank with accession numbers KM245931-KM245936.

### Acetylene reduction assay

Fore, mid and hindgut regions from wood- and mixed diet-fed fish were prepared as described above and divided in half, before being placed in either Hungate tubes containing 10 ml anaerobic nitrogen-free media or 2.0 ml gas chromatography (GC) vials (Perkin Elmer, Waltham, MA, USA) containing 500 μl of anaerobic nitrogen-free enrichment media. Tank water was obtained using a sterile syringe and 100 μl was used to inoculate enrichment media within sealed Hungate tubes and GC vials. Inoculated Hungate tubes were incubated at 25 °C for 48 h, shaking at 190 r.p.m. at a 45° angle, while samples placed in GC vials were immediately injected with 100 μl of acetylene, which was generated from calcium carbide (Postgate, 1972). GC vials were incubated at 25 °C without shaking prior to sampling at 1.5, 24, 48 and 72 h. To sample for acetylene reduction, 50 μl of headspace was drawn off using a gas-tight syringe and analyzed by a HP5890 gas chromatograph (Hewlett Packard, Palo Alto, CA, USA) equipped with a flame ionization detector and stainless steel column (0.32 × 182.88 cm) packed with silica gel (80/100 mesh; Supelco, Bellefonte, PA, USA). The column oven was operated at 110 °C with helium as the carrier gas. Purified ethylene (100 p.p.m.) was used as the standard (Supelco).

After 48 h, 100 μl of enrichment culture was transferred to a GC vial containing 400 μl fresh anaerobic media. Samples were immediately injected with acetylene and sampled at 1.5, 20 and 50 h post injection as described above. The OD_600_ was determined after 50 h using a Shimadzu UV-1601 spectrophotometer (Shimadzu Corp., Columbia, MD, USA).

### Catalyzed reporter deposition immunohistochemistry

GI tracts from wood-fed fish were divided into fore, mid and hindgut sections and immediately placed in 10 ml non-aqueous Carnoy's fixative (Matsuo *et al.,* 1997) for 2 h at 4 °C, transferred to 100% ethanol and stored at −20 °C. Tissues were equilibrated in a 30% sucrose solution at 4 °C overnight before being embedded in optimal cutting temperature compound (Sakura, Florence, CA, USA). Embedded tissues were frozen in a dry ice/ethanol bath and stored at −20 °C prior to sectioning. Tissues were serially sectioned at 12 μm at −25 °C using Accu-edge low profile blades (VWR, Radnor, PA, USA) on a Sakura Tissue-tek Cryo3 (Sakura Finetek, Torrence, CA, USA). Sections were immobilized on Superfrost Plus slides (VWR) and stored at 4 °C prior to staining.

Sections were permeabilized by applying 1 ml permeabilization buffer (12.0 mg lysozyme per ml, 50 mM EDTA in 100 mM Tris, pH 8.0) and incubating at 37 °C for 45 min. in a humidified chamber. Endogenous peroxidases were quenched with a 2% H_2_O_2_ solution for 5 min at room temperature in the dark. Samples were blocked (0.5% bovine serum albumin in phosphate-buffered saline) for 30 min at room temperature. Sections were incubated with a 1:500 dilution of primary antibody—chicken polyclonal IgY specific for KLH-conjugated synthetic peptides derived using NifH sequences from *Synechoccocus* sp. Q2JP78, *Trichodesmium theibautii*, *Anabaena* sp. P33178 and *Nostoc* sp. Q51296 by Agrisera PEB, Vannas, Sweden—in blocking solution for 1 h at room temperature. Slides were washed 3x with phosphate-buffered saline for 5 min before application of secondary antibody (anti-chicken IgY conjugated with horseradish peroxidase from Agrisera PEB). Secondary antibody was applied at a 1:1000 dilution (in blocking reagent) and incubated at room temperature for 1 h. Tyramide signal amplification (Perkin Elmer) was performed using Cy3 according to manufacturer's instructions. Tissues were counterstained with SYBR green (Life Technologies) for 2 min in the dark followed by a wash with dH_2_O for 1 min. Slides were dried with a reverse graded ethanol series and stored in the dark. Imaging was performed using a Zeiss Axioskop confocal microscope equipped with a Biorad Radiance 2100 laser scanning system at 476 and 543 nm. Images were manipulated using the Zeiss Laser sharp program.

### Sequence analysis and phylogenetic tree construction

Nucleotide sequences were trimmed automatically using a Phred quality score of 20 and sequences were pooled into operational taxanomic units (OTUs) using a 99% sequence identity threshold. Binned OTUs were used as search queries with the nucleotide Basic Local assignment Search Tool (BLASTn) and the highest scoring hit for each query was recorded. For phylogenetic reconstruction, the ~350 bp *nifH* DNA sequence was translated using the ExPASy bioinformatics resource tool (Gasteiger *et al.,* 2003) and amino acid sequences were used as search queries using the NCBI protein BLAST(BLASTp) (Altschul *et al.,* 1990), collecting the highest scoring match for each sequence. For tree construction, a single amino acid change was considered to be a unique OTU. Alignments were performed automatically using MEGA5 (Tamura *et al.,* 2011) followed by manual editing. Trees were constructed in MEGA5 using the neighbor joining method with 1000 bootstrap resamplings using default settings.

## Results

### nifH diversity

**Figure 1:**
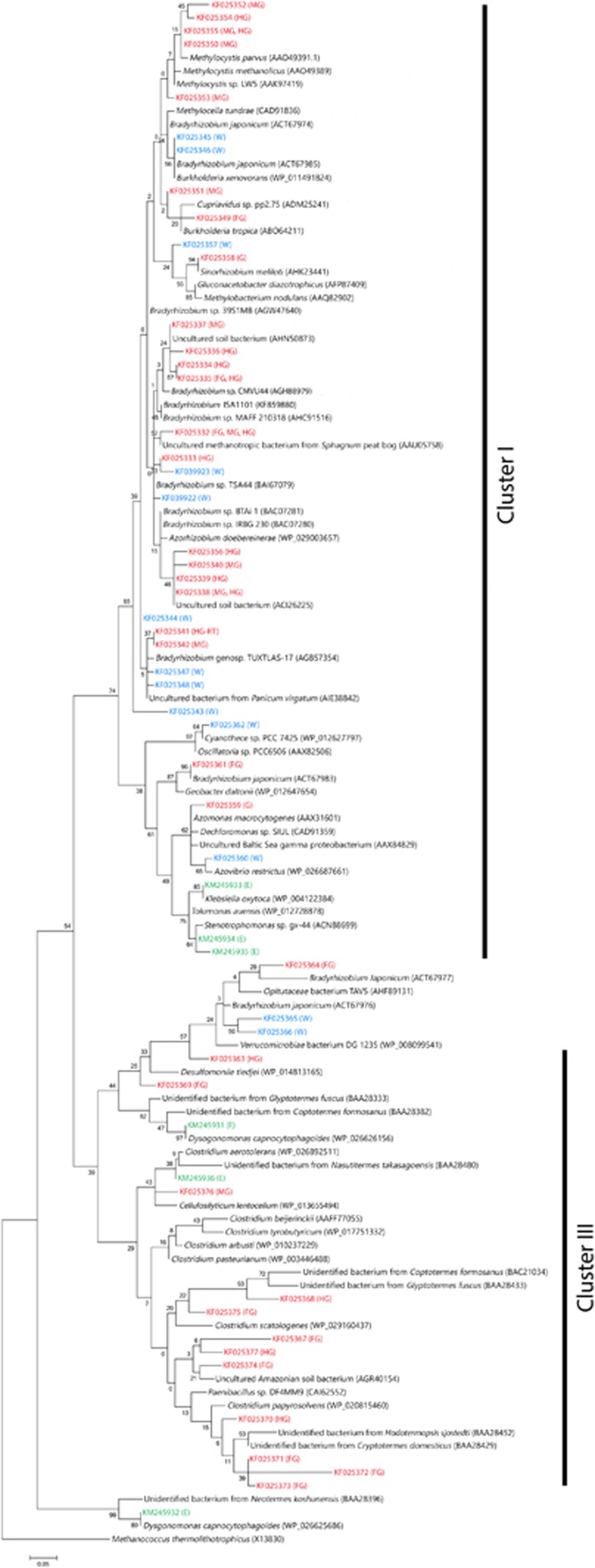
*nifH* phylogenetic tree. Phylogenies were reconstructed from *nifH* amino acid sequences recovered from culture water (W), entire GI tracts (G), enrichment cultures (E), and fore (F), mid (M) and hindgut (H) regions. Phylotypes from GI tract regions are indicated in red, enrichment cultures in green and culture water in blue. The tree was constructed as described in the Materials and methods section.

Nitrogenase genes were amplified from *P. nigrolineatus* GI tract regions and tank water using nested PCR and universal primers for the dinitrogenase reductase structural gene (*nifH*) as described in the materials and methods section. Phylogenetic relationships of *nifH* DNA sequence and *nifH*-deduced amino acid sequences were determined and results are shown in Table 1 and Figure 1, respectively. Out of ~250 clones obtained from GI tract and system water samples, 25 unique *nifH* sequences were obtained. The largest collection of sequences was distributed within the Cluster I *nifH* group (Chien and Zinder, 1996; Zehr *et al.,* 2003), which includes bacterial Mo-Fe nitrogenase operons and contains *nifH* sequences from Alpha-, Beta- and Gammaproteobacteria. Within this cluster, the closest relatives to the *nifH*-deduced amino acid sequences in GenBank were the symbiotic nitrogen-fixing *Bradyrhizobium* and *Sinorhizobium* spp. and the free-living nitrogen-fixing *Methylocystis*, *Gluconactobacter*, *Axovibrio* and *Azomonas* spp.

Representatives were also found among the Cluster III *nifH* group (Chien and Zinder, 1996; Zehr *et al.,* 2003) that includes *nifH* genes from Deltaproteobacteria, the Firmicutes and uncultured representatives from termite guts. Cluster III *nifH* sequences from GI tract samples were most closely related to *Clostridium*, *Cellulosilyticum* and *Desulfomonile* spp. In addition, three *nifH* sequences were found to be highly similar to those identified from the gut microbiota of the lower termites *Hodotermopsis*, *Cryptotermes* and *Coptotermes* spp., which depend on a symbiotic relationship between microbial communities and resident gut protists for wood digestion and nitrogen fixation (Ohkuma, 2008).

With the exception of one *nifH* phylotype that displayed high sequence similarity to Betaproteobacterium *Azovibrio restrictus*, *nifH* sequences identified from tank water (designated with a ‘W' in Figure 1 and Table 1) were similar to those found in the GI tract. However, many sequences were unique to the *Panaque* GI tract and not detected in the tank water, including the Clostridiales, and sequences related to those identified in termite GI tracts. The prevalence of *nifH* sequences along the GI tract did not appear to be localized as they were found distributed along fore, mid and hindgut regions.

### nifH gene expression

**Figure 2:**
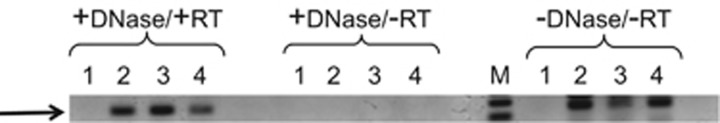
RT–PCR amplification of *nifH* mRNA from wood-fed *P. nigrolineatus* GI tract regions. After dissection, RNA was extracted from fore (lane 2), hind (lane 3) and midgut (lane 4) regions, as described in the materials and methods section. RNA (3 μg) was first treated with DNaseI in the presence of RNasin and RT–PCR was carried out using the Superscript III One-Step Platinum *Taq* high fidelity kit (see Methods). Complementary DNA synthesis was followed by amplification with nifH32 and nifH623 primers and a second round amplification using nifH1 and nifH2 primers as described in the materials and methods section. Reaction products were separated on a 1.5% agarose gel. RNA preparations treated with DNase in the absence of RT and amplifications without DNase and RT treatments are shown. M is a 100 bp ladder; lane 1 is a negative control that did not contain RNA.

The production of *nifH* messenger RNA (mRNA) in GI tract microbial communities was examined using RT–PCR as described in the materials and methods section and results are shown in Figure 2. No amplification was detected in RNA samples from fore, mid or hindgut regions in the absence of the RT reaction step (Figure 2), while amplification products were detected from these regions in the presence of RT corresponding to ~350 bp-sized fragment. While multiple attempts to clone fragments generated by RT–PCR *nifH* complementary DNA amplification from foregut and midgut regions were unsuccessful, the hindgut region yielded 22 clones. This clone collection was found to contain a single unique sequence with 99% DNA sequence similarity to *Bradyrhizobium* sp. (KF024341; Figure 1 and Table 1), whose sequence was also identified from the midgut *nifH* DNA library (KF025342; Figure 1). Thus, at least one *nifH* phylotype detected in the *P. nigrolineatus* GI tract is actively expressing *nifH in situ*, suggesting that the environment supports nitrogen fixation.

### Immunohistochemical analysis of nitrogenase in GI tract samples

**Figure 3:**
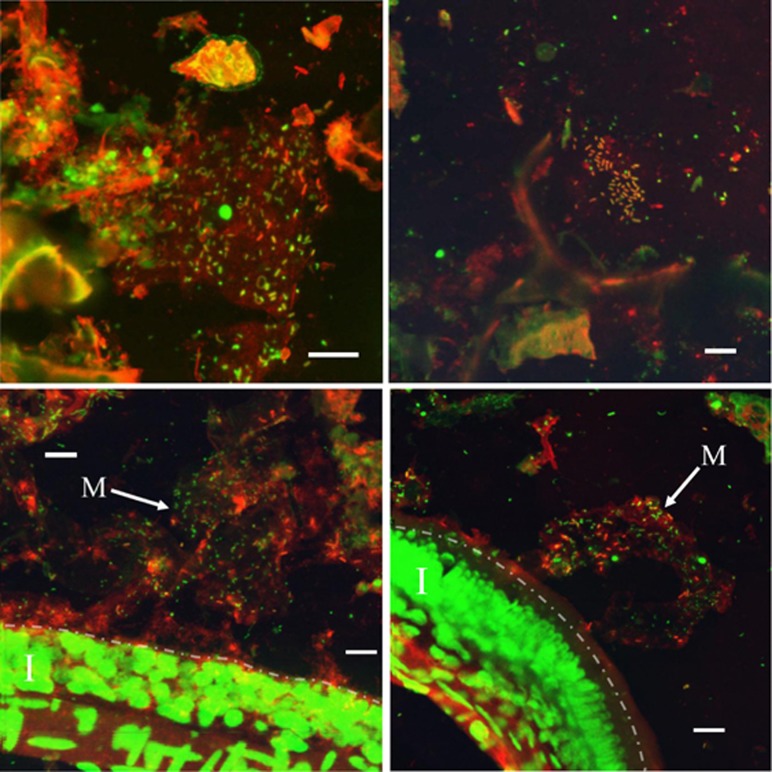
Distribution of nitrogenase-containing cells visualized via confocal laser microscopy. GI tract tissues were fixed in non-aqueous Carnoy's solution and embedded in paraffin as described in the materials and methods section. Semi-thin sections (3–5 μm) were visualized through catalyzed reporter deposition with anti-nitrogenase antibodies conjugated with horseradish peroxidase. Cells were counterstained with SYBR green. Green color denotes positively stained SYBR green tissues and cells, while red represents regions of catalyzed reporter deposition. Top panels: intestinal lumen regions containing nitrogenase-positive cells associating within and around wood fragments. Bottom panels: high-density nitrogenase-positive cells within the mucosal matrix (M) layer that had detached from the intestinal (I) wall (outlined by the dotted and dashed line). Bar, 10 μm.

The presence of nitrogenase-containing cells within the lumen of the GI tract was verified through catalyzed reporter deposition immunohistochemical analysis as described in the materials and methods section and typical photomicrographs are shown in Figure 3. Staining with a general nucleic acid stain (green in Figure 3) revealed an extensive microbial community extending through the entire length of the GI tract, which agreed with our previous finding (Watts *et al.,* 2013). Microbial cells were observed colonizing both the epithelial surface embedded within a mucosal matrix (‘M' in Figure 3, bottom panels) and attached to digesta as well as free-living within the lumen (Figure 3, top panels). Staining with anti-NifH antibodies revealed a smaller, irregularly distributed spherical and rod-shaped diazotrophic community with localized regions of increased density (red cells in Figure 3). Anti-NifH-stained cells were predominantly observed attached to digesta or epithelial surface, with relatively fewer free-living in the lumen. Colonization of the epithelial surface by nitrogenase-containing cells was observed only in the mid and hindgut regions. In contrast, woody digesta from all tissue regions was shown to possess anti-NifH-stained cells, suggesting that diazotrophic organisms may be entering the GI tract via ingested wood and, perhaps, colonizing the mid and hindgut regions of the intestines. Staining with secondary antibodies alone did not result in any signal, indicating that cross-reacting material is not a result of non-specific binding (not shown).

### Examination of enrichment cultures from nitrogen-free media

To examine the culturable nitrogen-fixing microbial community, tissue samples from wood-fed fish were harvested under anaerobic conditions and placed into Hungate tubes containing nitrogen-free enrichment medium as described in the materials and methods section. After two passages of growth for 4 days, cultures were harvested and *nifH* genes were cloned and sequenced (Table 2 and Figure 1). Although sequence diversity was less than that obtained directly from the GI tract, which is likely due to the selective nature of the enrichment medium, phylotypes were obtained from both Cluster I and Cluster III *nifH* sequences. Cluster I included Gammaproteobacteria related to *Klebsiella oxytoca* and S*tenotrophomonas* sp., two genera that include endophytic diazotrophs (Jha and Kumar, 2007; Ramos *et al.,* 2011) associated with grasses and sugar cane, respectively. Cluster III included a phylotype similar to *Clostridium* sp. (KM245936), which is similar to the one found in the midgut (KF025376) (Table 2), and related to an OTU identified from *Nastutitermes takasagoensis*, a lower termite species (Figure 1). The Cluster III *nifH* sequences were classified as Bacteroidia with sequence similarities to *Dysgonomonas capnocytophagoides* (Table 2), and related to unidentified bacteria in the lower termite *Coptotermes formosanus* (Figure 1). A phylotype that was not associated with either Cluster I or III was also detected that had high similarity to another *D. capnocytophagoides nifH* gene sequence and was related to an unidentified bacterium from the lower termite *Neotermes koshunensis* (Figure 1).

### Acetylene reduction assays of GI tract enrichment cultures and tissues

The conversion of acetylene to ethylene has been used as an indicator of nitrogen-fixing activity for both terrestrial and aquatic systems (Myrold, 2007) and we used this assay to examine for the presence of nitrogenase activity in enrichment cultures prepared from GI tract samples as described in the materials and methods section. Ethylene production was detected in fore, mid and hindgut enrichments from wood-fed fish with accumulation rates ranging from 4.0–22 mM ethylene per min per OD_600_. For fish raised on a mixed diet, activity was detected in the foregut enrichments exclusively, with an accumulation rate of 28 mM ethylene per min per OD_600_.

Since nitrogenase activity was detected in nitrogen-free enrichment cultures from the GI tract, as well as identifying *nifH* mRNA and antigen, GI tract tissues were directly analyzed for nitrogenase activity using the acetylene reduction assay. Tissue samples from wood-fed and mixed-diet treated fish were placed into GC vials under anaerobic conditions and ethylene production from acetylene was measured as described in the materials and methods section. Ethylene production was detectable in the fore and midgut of wood-fed fish, ranging from 11–15 ng per day per g, as well as the midgut and hindgut of mixed diet fish, varying between 9–84 ng per day per g. Activity was also detectable in the water with a production rate of 42 ng per day per g. These results support our finding that the *P. nigrolineatus* GI tract microbial community possesses nitrogen fixation activity and that the GI tract environment is conducive for this activity.

## Discussion

Symbiotic diazotrophic microbial populations have an essential role in primary consumers of lignocellulosic biomasses. These communities are capable of supplying the host with reduced nitrogen compounds, thereby closing or reducing the nitrogen deficit that occurs when consuming a diet rich or exclusively composed of vegetal or woody materials. Traditionally, most research on diazotrophic symbioses within vertebrate hosts has focused on ruminant animals, despite fish comprising the vast majority of global vertebrate diversity (Wilson and Reeder, 2005; Nelson, 2006). In the present study, the enteric diazotrophic microbial population of *P. nigrolineatus* was characterized through a combination of culture-dependent and -independent methods, providing insight into their potential impact on fish nutrition. To our knowledge, this is the first study on microbial nitrogen fixation occurring in a fish.

Several *nifH* phylotypes were identified in all tissue regions of the *P. nigrolineatus* GI tract, with representatives found from *nifH* Clusters I and III. Most Cluster I phylotypes had greatest sequence similarity to Alphaproteobacteria, however, Gammaproteobacteria-related species were also present. The Alphaproteobacteria were almost invariably members of the order Rhizobiales and included *Rhizobium*, *Bradyrhizobium, Methylocella* and *Methylocystis* species. The majority of Cluster III phylotypes were identified as either Clostridiales or unidentified enteric isolates similar to those found in lower termites. These findings are consistent with our previous 16S rRNA gene analysis that showed a high proportion of Rhizobiales and Clostridiales in the mid and hindgut regions (McDonald *et al.,* 2012; Watts *et al.,* 2013).

Comparisons across tissue regions revealed differing distributions of the *nifH* clusters. Cluster I sequences were ubiquitous and recovered from all tissue regions including the tank water. This contrasts to Cluster III sequences that were recovered predominantly from tissues or enrichment cultures. This may be due, in part, to the differing physiologies of Clusters I and III microorganisms; Cluster I includes both aerobes and facultative anaerobes while Cluster III are typically strict anaerobes (Zehr *et al.,* 2003). Unlike our previous 16S rRNA gene analyses (McDonald *et al.,* 2012), the tank water environment did not exhibit the greatest *nifH* diversity; a single OTU similar to *Cyanothece* sp. was identified in the tank water exclusively. This strongly suggests the diazotrophic population colonizing the GI is unique relative to the environment and likely does not represent a transient population.

Comparisons of clone libraries established from enrichment cultures and DNA extracted directly from tissue revealed different community profiles. Although both libraries were predominantly comprised of sequences most similar to Alphaproteobacteria, the specific species varied. These results are consistent with other studies that utilized enrichment cultures and direct cloning to characterize communities in the same environmental sample (Chelius and Triplett, 2001; Smith *et al.,* 2001; Nicol and Prosser, 2011). Differences in community composition arise from the selective nature of the enrichment culture, which may allow numerically minor species to outcompete predominant species.

Meta analyses of several teleost microbiomes revealed that clostridial species were enriched in GI tracts of herbivorous species, where they are thought to play a central role in cellulose degradation and fermentation (Sullam *et al.,* 2012). The order Clostridiales contains several well-characterized and prolific diazotrophic species (Dilworth, 1966; Chen *et al.,* 2001). These species are predominantly known to fix nitrogen as free-living organisms, but have, on occasion, been shown to fix nitrogen in association with a host organism including plants and termites (Ohkuma *et al.,* 1999; Levy-Booth *et al.,* 2014). Phylogenetic analysis of *nifH* sequences recovered from *P. nigrolineatus* revealed several OTUs sharing high sequence similarity with putative Clostridales identified in GI tracts of several termite species. The sequences, KF025368 and KF0255375, were predominantly associated with termite Clusters I and II with a few OTUs most closely related to termite pseudo-Nif cluster I, which resemble *nifH* based on sequence similarity, but, likely does not encode functional nitrogenases (Ohkuma *et al.,* 1999; Noda *et al.,* 2002).

The prevalence of Rhizobiales in all tissue regions is intriguing given their ability to fix nitrogen both in the free-living state and in association with plant hosts (Keyser *et al.,* 1982; Dreyfus *et al.,* 1983), although they are rarely identified as a major constituent of enteric microbiomes. Rhizobiales have been identified in the GI tract of few fish—including *P. nigrolineatus* (McDonald *et al.,* 2012; Watts *et al.,* 2013)—and termite species through both culture-dependent (Fröhlich *et al.,* 2007) and -independent analyses (Wu *et al.,* 2010), but their role in nutrient acquisition *in vivo* is unclear. Members of the arboreal ant genus *Tetraponera* have been shown to harbor root nodule-associated communities that included several species of Rhizobiales (*Rhizobium* and *Bartonella*) (Van Borm *et al.,* 2002). Like *P. nigrolineatus* growing on a wood-only diet, the diet of *Tetraponera* ants is rich in carbohydrates, but, greatly limited in nitrogen. While *nifH* sequences have been amplified from the gut of *Tetraponera* ants, it is still unclear whether nitrogen fixation is occurring, and these sequences may represent bacterial populations that are recycling nitrogenous waste and not fixing nitrogen *de novo* (Stoll *et al.,* 2007).

Our findings indicate that the GI tract environment of *P. nigrolineatus* is conducive for nitrogenase gene expression; a single *nifH* RT product demonstrating high sequence similarity to *Bradyrhizobium* sp. *nifH* was obtained from the hindgut region and was identical to sequences recovered from the midgut library. The detection of a single RT product in the hindgut is unexpected given the diversity of *nifH* phylotypes identified through the entire GI tract. Unlike well-characterized xylophagic organisms, the *P. nigrolineatus* GI tract is largely morpho-physiologically undifferentiated along its entire length. There is a lack of an extended hindgut or cecum, which is generally the primary location for nitrogen fixation in other organisms.

We believe that the *nifH* mRNA detected in the hindgut was likely the product of the resident microbial population in the gut microenvironment rather than due to transient communities consumed from the tank water. While nitrogenase mRNA is remarkably stable (20–30 min half-life) (Kahn *et al.,* 1982), the presence of oxygen reduces its stability significantly (approx. 5 min half-life) (Collins *et al.,* 1986) and the tank water from which these transient communities are originating would be highly aerated and not conducive to *nif* expression. Furthermore, while we were not able to detect *nifH* expression in the tank water microbial community (not shown), any *nifH* mRNA produced by nitrogen-fixing microorganisms that may have been consumed by the fish would have been detected throughout the GI tract, yet we could only detect an RT product from the hindgut community.

It is unclear whether detection of one phylotype *nifH* transcript in the hindgut represents the general pattern of nitrogenase expression within the GI tract. Amplification of *nifH* transcripts was challenging as the degenerate primer set used for amplification from total RNA also amplified a small (~350 bp) region of the *P. nigrolineatus* 18S rRNA (Schreier, unpublished) and off-target sequence amplification is not uncommon using degenerate, nested, *nifH* primers that have high coverage (Gaby and Buckley, 2012). Furthermore, the environmental matrix from which RNA was extracted provided unique challenges. The palm wood fed to fish is high in polyphenolic compounds, which have been shown to inhibit polymerase activity (Opel *et al.,* 2010). Given the diversity of *nifH* phylotypes present in the initial clone libraries, it is likely that deeper sequencing methods would have revealed greater *nifH* transcript diversity.

In addition to detecting *nifH* mRNA, NifH protein was identified within the gut lumen. Immunostaining of sectioned tissues revealed the presence of nitrogenase-containing cells attached to woody digesta within the lumen in all tissue regions as well as the mucosal matrix in the mid and hindguts. These findings are interesting given the low binding affinity of *Rhizobium* for fish intestinal mucus (Namba *et al.,* 2007). The presence of nitrogenase-containing cells in the digesta of the foregut without any positively stained cells attached to the epithelium mucosa in this region suggests the diazotrophic community is entering the GI tract from the environment and possibly entering symbiostasis in the mid and hindgut regions, perhaps in a manner similar to other animal-bacterial interactions (McFall-Ngai, 2014) and benefitting from that interaction (Berry *et al.,* 2013). This notion is further supported by the fact that the same *nifH* OTUs were identified in the tank water as the GI tract. Our previous analysis of the enteric community of *P. nigrolineatus* revealed the highest microbial diversity in the tank water while each of the tissue regions were enriched for specific subsets of that community (McDonald *et al.,* 2012). The lack of a well-defined acidic stomach in *P. nigrolineatus* (Schaefer and Stewart, 1993) may allow diazotrophic microorganisms to survive passage into the GI tract where they can colonize the hindgut mucosa (Pérez *et al.,* 2010).

Additional evidence for nitrogen fixation *in vivo* was obtained by acetylene reduction assays performed using freshly dissected GI tract tissue. Fixation rates were highly variable across tissue regions with no discernable difference in activity between feeding regimens. While these rates were lower than those typically observed in xylivorous insects (Pandey *et al.,* 1992; Berry *et al.,* 2013) they are consistent with those observed in herbivorous and granivorous grouses (Vecherskii *et al.,* 2014) and gerbils (Kuznetsova *et al.,* 2010), respectively. The relatively low activity rates may be due, in part, to the nature of the sampled tissues. Studies that have characterized nitrogen-fixing potential of enteric communities have typically examined whole, live specimens, or digesta that had been removed from the GI tract (Pun and Satter, 1975; Prestwich and Bentley 1981; Ohkuma *et al.,* 1999). Assays using live animals were not possible and the *P. nigrolineatus* GI tract is extremely long and thin-walled making extraction of digesta difficult under anaerobic conditions. As a result, intact sections of GI tract were assayed directly. Consequently, it is possible that after dissection and during incubations, degradation of GI tract tissue may have provided a significant pool of reduced nitrogen, which would directly reduce nitrogen fixation rates. Despite this, we were able to detect significant ethylene production from acetylene thereby providing further evidence for the ability to fix nitrogen *in vivo*. To our knowledge, this is the first demonstration of nitrogenase activity from the intestines of a fish species.

While the identification of *nifH* mRNA, immunostaining and acetylene reduction assays confirmed the presence of nitrogenase-producing cells in the GI tract, these approaches alone do not definitively validate that the enteric diazotrophic community is capable of fixing nitrogen *in vivo*. In many free-living diazotrophs, nitrogenase activity is inhibited by posttranslational ribosylation in response to excess nitrogen or limited energy availability (Nordlund and Högbom, 2013) and nitrogenase activity can be irreversibly terminated upon exposure to O_2_ (Robson and Postgate, 1980). Although the majority of *P. nigrolineatus* GI tract is anaerobic, studies in termites have shown that the gut epithelial surface can serve as a major source of molecular oxygen. The diffusion of oxygen from the epithelium can generate a steep oxygen gradient that can extend into the lumen where it can influence community composition and metabolism (Tholen and Brune, 1999; Brune and Friedrich, 2000).

Microscopic analysis of intestinal tissue and *nifH* cloning was consistent with the presence of a stable, resident microbial diazotrophic population and supported our earlier findings based on 16S rRNA gene analysis (McDonald *et al.,* 2012). Not surprisingly, the *P. nigrolineatus* enteric microbial community has been found to shift as a consequence of dietary conditions, with a more diverse cellulolytic population appearing as the diet becomes exclusively wood based (Watts, McDonald and Schreier, in preparation). However, *nifH*-containing populations are always identified even when fish were provided food rich in reduced nitrogen, suggesting a selective pressure of the gut environment for organisms at a functional level by an unknown mechanism.

The prospect of nitrogen fixation within the gut of teleosts could have major impact on our understanding of nitrogen fluxes in aquatic environments. In most stream environments, nitrogen is a major factor limiting primary production, biomass increases and nutrient content of primary producers (Peterson and Grimm, 1992; Stelzer and Lamberti, 2001). This is particularly true for environments where allochthonous carbon (leaves, woodfalls and so on) is the primary carbon source or in regions, like the Amazonian flood plain, that undergo periodic drought events where there is limited nutrient input (Bernhardt and Likens, 2002; Pinay *et al.,* 2002).

The results of this study provide the first direct evidence for nitrogen fixation processes within the GI tract of a teleost species, *P. nigrolineatus*. Through a combination of molecular, immunological and biochemical assay techniques it was demonstrated that the gut environment is conducive for *nifH* transcript expression and protein activity. These findings are consistent with those from well-characterized xylivorous systems and further demonstrate the importance of diazotropic populations in wood-feeding organisms. Analysis of the enteric microbial community revealed several OTUs with high sequence similarity to Rhizobiales, although the prolific nitrogen fixers Rhizobiales do not typically form animal associations and are rarely observed as members of fish microbiomes. The prevalence of Rhizobales in the GI tract of *P. nigrolineatus* may demonstrate the potential significance of ‘nontraditional' host microbe interactions and may expand our understanding of the diversity of symbioses occurring in nature. Metatranscriptomic and metabalomic analyses that focus on the nature of these interactions are ongoing.

## Figures and Tables

**Table 1:** Sequence similarity of the *nifH* gene amplified from system water and *P. nigrolineatus* GI tract samples^a^

*Class*	*Phylotype detected (source)*	*Closest GenBank Species (accession)*	*Count*^b^	*% Similarity*
Alphaproteobacteria	KF025332 (FG, MG, HG, W)	*Bradyrhizobium* sp. TSA44 (BAI67079)	7	96–99
	KF025338 (MG, HG)	*Bradyrhizobium* sp. BTAi 1 (BAC07281)	3	96–98
	KF025345 (FG, W)	*Bradyrhizobium japonicum* (ACT67985)	4	98–99
	KF025363 (HG)	*B. japonicum* (ACT67976)	1	91
	KF025341 (MG, HG-RT)	*Bradyrhizobium* sp. MAFF 210318 (BAC07283)	2	99
	KF025337 (MG)	*Bradyrhizobium* sp. CMVU44 (AGH88979)	1	97
	KF025351 (MG)	*Bradyrhizobium* sp. BRUESC119 (AGO66000)	1	98
	KF025343 (W)	*Azorhizobium doebereinerae* (ACI46147)	1	92
	KF025358 (G)	*Sinorhizobium meliloti* (AHK23441)	1	100
	KF025349 (FG, MG, HG)	*Methylocystis parvus* OBBP (AAO49391)	5	96–98
	KF025356 (HG)	*Methylocystis* sp. LW5 (AAK97419)	1	97
	KF025357 (W)	*Gluconacetobacter diazotrophicus* (AFP87409.1)	1	94
	KF025336 (W, HG)	*Sphingomonas azotifigens* (BAE71134)	4	97–99
Betaproteobacteria	KF025360 (W)	*A. restrictus* (WP_026687661)	1	98
Gammaproteobacteria	KF025359 (G)	*Azomonas macrocytogenes* (AAX31601)	1	96
Deltaproteobacteria	KF025369 (FG)	*Desulfomonile tiedjei* (WP_014813165)	1	90
Clostridia	KF025367 (FG)	*Clostridium beijerinckii* (AAF77055)	1	89
	KF025375 (FG)	*C. arbusti* (WP_010237229)	1	91
	KF025370 (HG)	*C. papyrosolvens* (WP_020815460)	1	94
	KF025377 (HG)	*C. tyrobutyricum* (WP_017751332)	1	88
	KF025376 (MG)	*Cellulosilyticum lentocellum* (WP_013655494)	1	93
Opitutae	KF025364 (FG, W)	*Opitutaceae* bacterium TAV5 (AHF89131)	3	92–94
Unidentified Termite Bacteria	KF025374 (FG)	Unidentified bacterium from *Hodotermopsis sjostedti* (BAA28452)	3	91–92
	KF025372 (FG)	Unidentified bacterium from *Cryptotermes domesticus* (BAA28429)	1	92
	KF025368 (HG)	Unidentified bacterium from *Coptotermes formosanus* (BAC21034)	1	87

Abbreviations: BLAST, basic local assignment search tool; FG, foregut; G, gut; HG, hindgut; MG, midgut; OTU, operational taxanomic units; RT, reverse transcribed product; W, water.

a Comparisons were done based on DNA sequence using BLAST as described in Materials and methods.

b Number of unique OTUs sharing closest GenBank species.

**Table 2:** Sequence similarity of the *nifH* gene amplified from *P. nigrolineatus* GI tract enrichment cultures^a^

*Class*	*Phylotype detected*	*Closest GenBank species (accession)*	*% Similarity*
Bacteroidia	KM245931	*Dysgonomonas capnocytophagoides* (WP_026626156)	99
	KM245932	*Dysgonomonas capnocytophagoides* (WP_026625686)	98
Gammaproteobacteria	KM245933	*Klebsiella oxytoca* (WP_004122384)	100
	KM245934	*Stenotrophomonas* sp. gx-44 (ACN88699)	97
	KM245935	*Stenotrophomonas* sp. gx-44 (ACN88699)	95
Clostridia	KM245936	*Clostridium aerotolerans* (WP_026892511)	99

Abbreviations: BLAST, basic local assignment search tool; GI, gastrointestinal.

a Comparisons were done based on DNA sequence using BLAST as described in the Materials and methods section.
